# Effect of Omega-3 Fatty Acids on Telomeres—Are They the Elixir of Youth?

**DOI:** 10.3390/nu14183723

**Published:** 2022-09-09

**Authors:** Magdalena Ogłuszka, Paweł Lipiński, Rafał R. Starzyński

**Affiliations:** 1 Department of Genomics, Institute of Genetics and Animal Biotechnology of the Polish Academy of Sciences, 05-552 Jastrzębiec, Poland; 2 Department of Molecular Biology, Institute of Genetics and Animal Biotechnology of the Polish Academy of Sciences, 05-552 Jastrzębiec, Poland

**Keywords:** telomere, senescence, omega-3 fatty acid, oxidative stress

## Abstract

Telomeres are complexes consisting of tandem repeat DNA combined with associated proteins that play a key role in protecting the ends of chromosomes and maintaining genome stability. They are considered a biological clock, as they shorten in parallel with aging. Furthermore, short telomeres are associated with several age-related diseases. However, the variability in telomere shortening independent of chronological age suggests that it is a modifiable factor. In fact, it is regulated inter alia by genetic damage, cell division, aging, oxidative stress, and inflammation. A key question remains: how can we prevent accelerated telomere attrition and subsequent premature replicative senescence? A number of studies have explored the possible impact of omega-3 fatty acids on telomere shortening. This review summarizes published cross-sectional studies, randomized controlled trials, and rodent studies investigating the role of omega-3 fatty acids in telomere biology. It also covers a broad overview of the mechanism, currently favored in the field, that explains the impact of omega-3 fatty acids on telomeres—the food compound’s ability to modulate oxidative stress and inflammation. Although the results of the studies performed to date are not consistent, the vast majority indicate a beneficial effect of omega-3 fatty acids on telomere length.

## 1. Telomeres

Who wants to live forever… maybe not forever, and not as immortals, but for a long time and in good health? Probably most of us. The question is how to reach this goal. One solution may be to reduce the attrition of the telomere. Telomeres are nucleoprotein structures strongly related to mortality and aging-associated diseases. The concept of telomeres was raised in the 1930s and 1940s in parallel by two scientists: Hermann Muller and Barbara McClintock. Based on their pioneering research, they postulated that chromosomes end with structures that are distinct from DNA breaks and provide protection from the processes that fuse broken chromosomes [1,2]. Muller was the first to name them telomeres from the Greek terms “telos” (end) and “meros” (part) [2]. Back then, they failed to foresee the implications that their findings/discoveries would have for molecular biology and genetics and thus our understanding of the mechanisms that govern biological aging.

In vertebrates, telomeres consist of highly conserved hexanucleotide repeats of G-rich sequences (TTAGGG) at the ends of eukaryotic chromosomes and their binding proteins [3]. Telomeric DNA is composed of long stretches of double-stranded DNA and the single-stranded G-rich 3′ terminus called the G-overhang, which invades the double-stranded telomeric DNA, forming a structure known as a t-loop. Generating a t-loop allows the telomere to fold into a closed configuration that protects the chromosome ends from being identified as DNA double-strand breaks; thus, cellular DNA damage response pathways or chromosome fusion are not activated [4,5]. The length of human telomeres ranges from 5 to 15 kilobases [6], while rodents possess much longer telomeres, even up to 150 kilobases [7]. Telomeres and the rest of the chromosome are separated by subtelomeres and interstitial sections [8]. Telomeres are composed of not only telomeric DNA but also associated proteins. Nucleosomal histones and shelterin complexes are attached to telomeric DNA. The shelterin complex (also known as the telosome) is composed of TRF1 (telomeric repeat binding factor 1), TRF2 (telomeric repeat binding factor 2), TIN2 (TRF1-interacting nuclear factor 2), Rap1 (repressor/activator protein 1), TPP1 (also known as adrenocortical dysplasia protein homologue), and POT1 (protection of telomeres 1). Shelterin proteins play a dual role: they protect telomeres from deleterious DNA damage response processes and regulate the accession of telomerase to telomere DNA [9].

Telomeres are shortened with each successive cell division [10]. During DNA replication, a semi-conservative process, each DNA strand creating the double helix acts as a template for making the complementary strand. DNA polymerase, Polα, can replicate DNA only in one direction, from the 5′ end to the 3′ end. Therefore, the leading strand is copied continuously with a single RNA primer in the direction of the advancing replication fork, whereas the lagging strand is reproduced discontinuously, which requires the annealing of multiple primers that elongate into short Okazaki fragments opposite to the replication fork. When replication is completed, the RNA primers are removed, the resulting gaps are filled with Polδ polymerase, and the Okazaki fragments are joined together by DNA ligase to form a continuous strand. The gap left by the degraded primer at the terminal end remains unfilled, which results in the loss of a short segment of DNA at the 5′ end of the lagging strand. This is called the “end-replication problem”, a notion proposed by Watson in 1972 [11]. Therefore, the role of telomeres—located at the ends of chromosomes that are exposed to replicative shortening—is to protect against the loss of valuable genetic information. Still, with each division, the cell becomes about 250 nucleotides shorter [12].

How does telomere length relate to senescence? The very first studies by Leonard Hayflick et al. [13] shed some light on this issue: they reported that human diploid cells age because they can only divide a certain number of times, a phenomenon called the Hayflick limit. Fibroblasts cultured by Hayflick stopped dividing after 50 ± 10 passages. The first scientist who connected the Hayflick limit to telomere shortening was Alexey M. Olovnikov [14,15]. He postulated that the number of cell divisions is determined by the telomere length. Indeed, once the telomeres are shortened beyond a critical length, the shelterin complex is unable to associate with the telomeric sequence. Telomeres lose their protective structure and are recognized as double-strand DNA breaks, which induces a DNA damage response, stimulating ATM kinase that activates p53, which in turn causes G1 cell cycle arrest and aging [16,17,18]. Thus, telomeres act as a “mitotic clock” [19]. The accumulation of senescent cells is related to a reduction in the number of mitotic cells, leading to a reduced potential for tissue growth and repair. Moreover, accumulated senescent cells result in a senescence-associated secretory phenotype, which is based on the release of proteases, growth factors, and inflammatory cytokines, which in turn has a negative feedback effect on non-senescent neighboring cells [20,21]. These processes influence the aging of tissues and, consequently, of the organism.

Telomere theory is predicated on the idea that telomere shortening triggers aging. Telomere attrition causes the cell senescence that leads to aging, largely through cell exhaustion, which reduces tissue regeneration capacity and the secretion of proinflammatory factors that can destroy the stem cell environment [21]. Moreover, studies in mice showed that the reversion of the function or length of telomeres improved the mice’s life span and that telomerase gene therapy delayed physiological aging and extended longevity in mice [22,23]. In addition, short telomeres are the natural limit of human life spans [24]. The degree of telomere shortening is proportional to the risk of death, as well as of age-related diseases, including, but not limited to, immune deficiency, cardiovascular disease, cancer, diabetes, depression, and cognitive impairment [9]. In the face of these facts, it is crucial to identify the factors that can avert excessive telomere shortening, which in turn could prevent premature aging or treat age-related diseases.

Population studies have shown that telomere length is a modifiable factor and not only related to chronological aging, as a number of lifestyle and dietary factors can modify telomere attrition. One factor inversely related to telomere length is chronic stress, both during the prenatal period [25] and childhood [26], as well as in adult life [27]. Depression [28], smoking [29], obesity [30], and alcohol consumption [31] also accelerate telomere attrition. Interestingly, dietary restriction [32] and increasing dietary antioxidants [33] protect against telomere shortening. In this context, omega-3 fatty acids are important dietary compounds that, due to their biochemical properties, may affect the biology of telomeres, which we will cover in this review.

## 2. Omega-3 Fatty Acids

Fatty acids are a family of lipids, which are generally aliphatic monocarboxylic acids. Particles containing two or more double bonds are termed polyunsaturated fatty acids (PUFAs) [34]. Based on the location of the first cis double bond in the aliphatic chain, when counting carbon atoms from the methyl end of the fatty acid, PUFAs can be divided into three main families: omega-3, omega-6, and omega-9 fatty acids.

Fatty acids are delivered to the organism as triglycerides and phospholipids or free fatty acids. Before their absorption in the small intestine, triglycerides and phospholipids have to be hydrolyzed by lipases. The absorption of the majority of fatty acids occurs in the small intestine, where bile salts incorporate fatty acids and other fat digestion products into micelles. Then, the micelles are absorbed through enterocytes. The average absorption of ingested fat varies from 85 to 95% [35]. The concentration and composition of blood lipids depend on both the dietary intake and biotransformation of fatty acids. Hepatic fatty acid desaturation and elongation pathways are necessary, particularly when dietary long chain fatty acids are inadequately supplied [36,37].

An important member of the omega-3 fatty acid family is α-linolenic acid (ALA), which is called an essential fatty acid. The main sources of ALA are oils, nuts, and seeds, e.g., flaxseeds and flaxseed oil, walnuts and walnut oil, soybeans and soybean oil, pumpkin seeds, rapeseed oil, and olive oil [38]. Omega-3 fatty acids are indispensable for the proper functioning of organisms; however, they cannot be synthesized de novo by humans and animals because of the lack of fatty acid omega-3 desaturase enzymes, which are necessary for inserting a cis double bond at the omega-3 position of a fatty acid. Other omega-3 fatty acids are delivered with food or are products of ALA elongation and desaturation. However, their rate of conversion is low. Healthy young men convert approximately 8% of dietary ALA to eicosapentaenoic acid (EPA) and up to 4% to docosahexaenoic acid (DHA) [39]. The beneficial effect of long chain fatty acids of a marine origin—such as EPA and DHA—on homeostasis and disease prevention has been widely studied [40].

Omega-3 fatty acids are not only a source of energy. They are major biologic regulators of proper growth and development as well as disease. A supplementation with EPA and DHA leads to their abundant content in plasma lipids, platelets, erythrocytes, leukocytes, colonic tissue, cardiac tissue, and other cells [41]. An increased cell and tissue omega-3 fatty acid content alters cell functioning through a number of mechanisms. One of these mechanisms is related to changes caused by the incorporation of omega-3 fatty acids into cell membranes. Due to the high conformational flexibility of omega-3 fatty acids’ acyl chains, they can affect the membrane’s physical properties, membrane fluidity, and the structure of lipid rafts, which may further modify protein function, transport, protein–protein interactions, vesicle budding, and fusion [42]. Omega-3 fatty acid derivatives also serve as messenger molecules. They are involved in signal transduction in the nervous system and act as local hormones, stimulating and maintaining various actions in animal organisms, i.e., inflammation processes, the regulation of the blood supplied to organs, and ion transport through membranes [43]. Another regulatory function of omega-3 fatty acid pathways is related to the modification of gene expression. This regulation is based inter alia on modulating the expression of transcription factors, including sterol-regulatory-element-binding proteins (SREBPs) and peroxisome proliferator-activated receptors (PPARs) [44], as well as epigenetic alterations, such as histone modifications, DNA methylation, and the level of miRNAs associated with gene repression or activation [45]. Undoubtedly, omega-3 fatty acids play important and broad roles in the functioning of the body. Maintaining the optimal amount of and ratio between omega-3 fatty acids and other fatty acids in the diet helps to prevent diseases such as heart attacks, atherosclerosis, thrombosis, arrhythmia, stroke, immune-inflammatory disorders, asthma, arthritis, cancer, type II diabetes mellitus, obesity, and psychiatric disorders [46].

## 3. Omega-3 Fatty Acids and Telomeres

### 3.1. Human Studies

The cornerstone of studies on the impact of omega-3 fatty acids on telomere length was the work of Farzaneh-Far et al. [47]. They examined cohort studies of more than 600 patients with coronary artery disease (CAD), which showed strong evidence for an association between omega-3 fatty acid consumption and telomere length, and more precisely, an inverse relationship between the baseline blood levels of omega-3 fatty acids (DHA and EPA) and changes in leukocyte telomere length over five years. Studies by Cassidy et al. [48] focused on the impact of diet and lifestyle factors on telomere length and were published in the same year. The authors examined over 2000 women and estimated their PUFA levels on the basis of a lifestyle questionnaire. They found no significant association between omega-3 fatty acid intake and telomere length. The two newest cross-sectional studies, published by Chinese groups, are in line with the work of Farzaneh-Far et al. [47]. Chang et al. [49] examined 711 patients with nested CAD and 638 CAD-free controls. Using linear regression, they tested the association between omega-6 and omega-3 fatty acids and leukocyte telomere length. The plasma levels of omega-3 fatty acids, particularly EPA and DHA, were found to be positively correlated with telomere length. Similarly, a lower omega-6/omega-3 fatty acids ratio was significantly associated with longer telomeres. However, it should be highlighted that this correlation was mainly driven by elevated levels of omega-3 fatty acids, while omega-6 fatty acids, considered separately, had no effect on telomere length. The positive effect of omega-3 fatty acid content on telomere length has also been observed in obese children [50]. Forty-six 3- to 4-year-old preschool children with obesity were included in the study, with equal numbers of age- and gender-matched children of normal weight as controls. The obese children exhibited lower levels of DHA, and this parameter was positively correlated with a shorter leukocyte telomere length.

However, not all non-interventional studies are consistent. Several cross-sectional studies have found no effect of omega-3 fatty acid levels on telomere length. In addition to the above-mentioned publication by Cassidy et al. [48], Freitas-Simoes et al. [51] also reported no correlation. In the related study, elderly people from the Mediterranean region (63–79 years old; 68.6% women) were surveyed. In this group, no relationships were found among red blood cells’ ALA concentration, the combined level of EPA and DHA, and the length of leukocyte telomeres. Several factors can account for the radically different results from these cross-sectional studies. The fatty acid levels were estimated using gas chromatography, mass spectroscopy, or food-frequency questionnaires, and this diversity of approaches could result in discrepancies. Moreover, single samples collected to determine fatty acid levels may not reflect the subjects’ long-term fatty acid content over years, the importance of which cannot be overestimated when studying telomere biology. In addition, the variability between the examined groups was high. These variabilities were related to health status, age, sex, and nationality. Nevertheless, the results from a considerable number of non-interventional studies have indicated that omega-3 fatty acids play a role in telomere biology, which has also been investigated in randomized dietary studies.

An early randomized, controlled trial examining the effects of omega-3 fatty acids on telomere length involved 106 healthy, overweight, middle-aged, and elderly people (aged 40–85) [52]. In this double blind, four-month experiment, the participants were divided into three groups receiving: (1) 2.5 g/day omega-3 PUFAs, (2) l.25 g/day omega-3 PUFAs, or (3) placebo capsules that mirrored the proportions of fatty acids in the typical American diet. Omega-3 PUFAs were administrated as a 7:1 mixture of EPA:DHA; thus, the high-dose group received 2085 mg of EPA and 348 mg of DHA per day. The placebo was a mixture of palm, olive, soy, canola, and cocoa butter oils, with a saturated:monounsaturated:polyunsaturated fatty acid ratio of 37:42:21. Although the group differences with respect to telomere length were not significant, the changes in the omega-6/omega-3 plasma ratios were negatively correlated with telomere length, which suggested a positive effect of increasing omega-3 fatty acid content on cell aging. Although differences in telomere length between the groups were negligible after the omega-3 or omega-6 fatty acid administration, the changes in the plasma omega-6/omega-3 fatty acid ratios were negatively correlated with telomere length. Such a result suggested a positive effect of increasing the content of omega-3 fatty acids on cell aging. A year later, another pilot study was published, wherein O’Callaghan et al. [53] showed that telomere shortening in whole blood can be alleviated by a supplementation with omaga-3 fatty acids. In a 6-month intervention, the study’s authors observed greater telomere shortening in the participants given safflower oil containing mainly omega-6 LA (linoleic acid) than in the participants given omega-3 PUFAs, namely, the EPA group (supplemented with 1.67 g of EPA + 0.16 g of DHA/daily) and the DHA group (supplemented with 1.55 g of DHA + 0.40 g of EPA/daily). The limitation of this study might have been the size of the population (44 elderly individuals with mild cognitive impairments), which significantly weakened the strength of the statistical tests and the study’s conclusions. However, the strongest data from this study indicated that changes in telomere length were significantly associated with changes in DHA levels in red blood cells: those who experienced the greatest increases in red blood cell DHA levels showed the least degree of decrease in telomere length [53].

Supplementation with omega-3 fatty acids also affected leukocyte telomere length in patients with chronic kidney disease (CKD). In studies by Barden et al. [54], 85 patients suffering from CKD were divided into four groups and received omega-3 fatty acids (4 g), CoQ (200 mg), both supplements, or the control (4 g of olive oil) every day for 8 weeks. The capsules of omega-3 fatty acids contained 460 mg of EPA, 38 mg of docosapentaenoic acid (omega-3 DPA), and 380 mg of DHA. The intervention with omega-3 fatty acids was associated with an increase in neutrophil telomere length, which was corrected for the neutrophil count [54].

The research on the effects of prenatal omega-3 fatty acid supplementation on telomeres deserves a separate chapter. To the best of our knowledge, there are three major publications on this topic. The first two reported no impact of omega-3 fatty acids delivered during pregnancy on the telomere length of the offspring. See et al. [55] examined 98 atopic pregnant women randomly assigned to two groups—one given omega-3 fatty acids and the other olive oil as a control—from 20 weeks’ gestation until delivery. The omega-3 fatty acids were delivered in the form of 4 g fish oil capsules per day comprising a total of 3.7 g of omega-3 fatty acids with 56.0% containing DHA and 27.7% containing EPA. The supplementation did not change the offspring’s telomere length at birth or at age of 12, with no change over time. Similar results were obtained later by Yeates et al. [56] in cross-sectional studies involving 229 pregnant women. These studies compared the levels of omega-3 fatty acids in blood collected from mothers at 28 weeks of gestation and at childbirth and from their children at 5 years of age; the leukocyte telomere length was measured in the mothers at birth, as well as in the blood from the umbilical cord and in the blood of the children at age 5. The prenatal status of omega-3 fatty acids was not associated with maternal or infant telomere length nor with the rate of telomere shortening. In contrast, Liu and colleagues [57] arrived at the opposite conclusion. In their study, the mother’s blood before birth and the child’s blood after birth, as well as the blood from the umbilical cord and the placenta, were collected from 274 mothers and their children. Low concentrations of DPA and total omega-3 fatty acids in maternal erythrocytes, as well as low concentrations of cord blood DHA, were associated with shortened telomeres in the cord blood cells. Surprisingly, high concentrations of three omega-3 fatty acids—ALA, eicosatrienoic acid (EA), and DHA—in the maternal erythrocytes were associated with shortened telomeres in the placenta. The presented publications did not determine whether the maternal level/supplementation with omega-3 fatty acids affects the telomere length of the offspring. Discrepancies between the obtained results may be related to differences between the populations, the length and form of the fatty acids consumed, and, above all, the quality of life of the pregnant woman (Table 1).

### 3.2. Rodent Model Studies

To better understand the role that omega-3 fatty acids play in telomere biology, a number of studies have been carried out using rodent models (Table 2). In the first studies, various doses of omega-3 fatty acids were administrated to male mice for two months [59]. With the exception of the negative control group, all the mice received D-galactose to induce aging. The experimental groups were supplemented with 400, 200, and 100 mg of fish oil per kg of body weight per day or 120, 60, and 30 mg of DHA per kg of body weight per day. Compared to the aging model group, the animals that received high- and low-dose DHA showed a significant positive effect with respect to hepatic telomere shortening in the range of 13–25%, while a moderate dose of fish oil and low-dose DHA exhibited 25% and 27% inhibitory effects on testicular telomere attrition, respectively. Subsequent studies in a rat model confirmed the positive effect of omega-3 fatty acids on telomere length: 72 male rats were assigned to three groups and fed with fodder from different fat sources (virgin olive oil, sunflower oil, or fish oil rich in DHA and EPA) from weaning until 24 months [60]. The liver telomere lengths were measured in 6- and 24-month-old rats. Surprisingly, in the cited studies, the rats fed with a diet rich in omega-3 fatty acids showed not only a reduced rate of telomere attrition as described by Chen et al. [59] but also the elongation of their telomeres, as the hepatic telomeres of the 24-month-old rats were longer than those of the 6-month-old rats. The beneficial effect of omega-3 fatty acids on telomere length in rats was also reported by Gao et al. [61]. In their studies, the authors showed that the hepatic telomere length of the offspring suffering from gestational diabetes mellitus (GDM) was nearly improved (with a nonsignificant tendency) by a supplementation with omega-3 fatty acids compared with the non-supplemented GDM offspring. In a comparative study of telomere length, 11-month-old animals were divided into two diet groups: a control group (given 7% soybean oil) and an experimental group (in which 4% of the 7% soybean oil control diet was replaced with fish oil containing 60% omega-3 PUFAs (50% of DHA; 10% of EPA)). The experimental diet was administered starting from weaning. Thus, the studies carried out in rodents, which allow for well-controlled research and large amounts of omega-3 fatty acids supplementation in short-lived animals, confirm the positive effect of omega-3 PUFAs administration on telomere length.

## 4. The Role of Omega-3 Fatty Acids in the Modulation of Oxidative Stress and Inflammation: Involvement in the Telomere Theory of Aging

As stated in the introduction, telomeres are dynamic structures that protect the linear DNA at the ends of the chromosomes. As telomeres shorten with each stage of replication, the number of cell divisions over time is further limited. Therefore, telomeres are thought to serve as a “replicometer” for replicative senescence [62,63]. Not only do they trigger replicative senescence under conventional conditions, but they also act as sensors for cumulative oxidative stress by accumulating single-strand breaks and accelerating the shortening process. Extremely short telomeres can contribute to cell aging or lead to genomic instability and, consequently, contribute to many degenerative and age-related diseases, including cancer [64]. Cells that have lost their replicative capacity trigger senescence and the accumulation of age-secreting inflammatory cytokines, which promotes degenerative diseases and pathologies associated with aging [65]. However, precancerous cells lacking a functional p53 pathway bypass aging and continue to divide. Shortened ends are processed by DNA double-strand break repair machinery, leading to the chromosomal fusion and chromosomal instability that kills most cells, but that leads to malignant transformation in the survivors [66].

Oxidative stress refers to the excessive production of reactive oxygen species (ROS) in cells and tissues that the antioxidant system is not be able to neutralize. An imbalance in this protective mechanism can lead to the damage of cellular molecules, such as DNA, proteins, and lipids [67]. ROS are normally produced within the body and are important compounds involved in regulating the maintenance of cell homeostasis and functions such as signal transduction, gene expression, and receptor activation [68]. In addition, mitochondrial oxidative metabolism in cells produces ROS species and organic peroxides in the process of cellular respiration [69].

It has been suggested that telomere shortening and cellular senescence in the dividing cells during the aging process contribute to human diseases and pathologies, including cardiovascular diseases [70], autoimmune diseases [71], chronic obstructive pulmonary disease (COPD) [72], and metabolic disorders, such as diabetes. Often, these diseases display inflammatory parameters, underlining a strong link between telomeres and inflammation. Telomere length is considered an emerging biomarker for age-related diseases and oxidative stress, which can induce accelerated telomere shortening [73]. Overall, oxidative stress and inflammation are common features of various conditions and diseases in which patients have shorter telomeres compared to controls. Indeed, inflammation and oxidative stress are interconnected factors affecting telomere biology. Therefore, this situation is exemplary of the old dilemma about the chicken and the egg. Inflammatory oxidative stress leads to a massive production of ROS and reactive nitrogen species (RNS) that in turn cause extensive telomere attrition [74,75]. In addition, phagocytic immune cells, via the nicotinamide adenine dinucleotide phosphate (NADPH) oxidases, mediate oxidative stress, a major contributor to telomere damage, in their G-rich regions susceptible to oxidative damage [74,76,77]. Many experimental observations suggest that oxygen free radicals may somehow contribute to the aging process [78]. Several human population studies reported that markers for oxidative stress correlated with shorter average telomere lengths, as measured in white blood cells [79]. Similar correlations were observed in populations with higher perceived psychological stress and higher inflammatory loads [80,81]. Clearly, the role of ROS in aging needs to be put into context with respect to mechanisms that are known to be connected with life span, particularly the phenomenon of telomere shortening with age [82]. Several studies have been conducted showing that different ROS are involved in cell senescence and telomere attrition, but the mechanisms responsible for these processes are unknown. One possibility is that oxidative stress triggers cell death and/or senescence, and to compensate, the survivors undergo more cell divisions, leading to increased telomere shortening. Another widely cited model suggests that ROS induce single-strand breaks at telomeres directly, or as intermediates in lesion repair, leading to replication fork collapse and telomere loss [73]. Alternatively, lesions that impede telomere replication can cause an accumulation of unreplicated single-stranded DNA and manifest as multi-telomeric foci at chromatid ends termed ‘fragile telomeres’ [83]. Other possibilities are that oxidative lesions interfere with shelterin binding or its transcription at the telomeres into TERRA transcripts. Finally, the processing of oxidative lesions may lead to changes in the number of telomere repeats.

Many scientific studies point to ROS as the molecules responsible for telomere shortening. In a study using “young” human diploid fibroblasts treated with low doses of hydrogen peroxide (H_2_O_2_) over a prolonged period, Duan et al. [84] found that prolonged H_2_O_2_ treatment significantly accelerated the process of telomere shortening, indicating that prolonged oxidative stress caused by H_2_O_2_ can modify the rate of telomere shortening. Similar results have been obtained using chondrocytes from avascular tissues such as human articular cartilage. Prolonged moderate to acute oxidative stress (10–100 µM H_2_O_2_) considerably accelerated telomere shortening and cellular aging in these cells ex vivo. In addition, senescent chondrocytes showed a reduced tolerance to oxidative stress [85].

Another ROS-related factor is the superoxide anion (O_2_^●−^), whose production by the respiratory chain is estimated to be at rates somewhat less than 1% of the total rate of electron transport from NADH to oxygen [86]. The physiological fate of the hydrogen peroxide generated in the reaction of the dismutation of the two molecules of O_2_^●−^ on either side of the mitochondrial membrane by MnSOD or CuZnSOD (superoxide dismutase 1 or 2) is to be processed by glutathione peroxidase into water in a reaction that converts reduced glutathione to oxidized glutathione [87]. In a study by Kawanishi and Oikawa [88], it was shown that H_2_O_2_ plus a Cu(II) ion efficiently caused DNA damage at the 5′ site of 5′-GGG-3′ in the telomere sequence (5′-TTAGGG-3′). Using an SIN-1 donor simultaneously generating nitric oxide (^●^NO) and a superoxide anion (O_2_^●−^), the authors showed that these radical molecules efficiently caused DNA cleavage at the 5′ site of the telomere sequence. A similar DNA cleavage pattern was observed when a combination of a ^●^NO-generating agent (NOC-7) and an O_2_^●−^-generating system (BQ + NADH) was used. These data also show that nitric oxide may be one of the contributors to age-related oxidative stress. Supporting this idea is the research by Chou et al. [89] demonstrating reduced endothelial nitric oxide synthase (eNOS) expression with increasing age in rats. It is well documented that the capacity of the vascular endothelium to generate bioactive ^●^NO decreases with an advancing age even in healthy subjects, and this phenomenon may reflect a decreased expression of NO synthase, as well as an increased production of superoxide, which contributes to the increased vascular risk associated with aging [90]. Studies with cultured endothelial cells suggest that the rate of endothelial aging is determined primarily by the rate of cell turnover and the associated progressive shortening of telomeres [91]. Endothelial cells transfected with the catalytic subunit of telomerase—which preserves a youthful telomere length—did not show a reduction in NO synthase expression after numerous doublings, in contrast to the marked reduction observed in control cells [92]. The mechanism of the direct or indirect action of pro and anti-inflammatory factors on the biology of telomeres has not been sufficiently elucidated; however, many studies indicate such a possibility. The signaling downstream of inflammatory cytokines such as IFN-α plays an important role in the downregulation of TERT activity in hematopoietic cells [93]. In contrast, interleukin (IL-6) and tumor necrosis factor (TNF)-α reportedly upregulate TERT transcription and telomerase activity through the activation and binding of NF-κB in macrophages [94] or NF-κB, STAT1, and STAT3 interactions with the TERT promoter in splenocytes and cancer cells [95,96].

Regarding the telomere theory of aging, several recent studies, as shown in the first part of this review, have provided epidemiological evidence that omega-3 PUFAs may prevent excessive telomere attrition. Interestingly, the capacity of omega-3 fatty acids to protect telomeres is often attributed to their antioxidant and anti-inflammatory properties, which we will try to demonstrate below in light of the available research.

At the beginning of the 21st century, many studies indicated that telomerase activity and thus the maintenance of telomere length are highly regulated processes. A number of factors, such as ROS, nitric oxide, stress, hormones, growth factors, inflammation, socioeconomic status, lifestyle, and diet, have been suggested as putative telomere length modulators [89,97,98]. Thus, the pioneering work by Farzaneh-Far et al. [47]—indicating for the first time that among patients with stable CAD, there was an inverse relationship between the baseline blood levels of marine omega-3 fatty acids and the rate of telomere shortening over 5 years—contributed to the in-depth study of the role of omega-3 fatty acids and their effects on the aging of telomeres. Although the measurements were limited to the telomere length in leukocytes and did not necessarily reflect the telomere trajectory in other cellular compartments, such as the myocardium, endothelium, or atherosclerotic plaque, they showed that patients receiving omega-3 fatty acids had lower levels of C-reactive protein (CRP), low-density lipoprotein cholesterol (LDL-C), and IL-6, clearly demonstrating the anti-inflammatory role of DHA and EPA in coronary heart disease. Similarly, Farzaneh-Far et al., in their previous cross-sectional associations, showed that omega-3 fatty acid levels were inversely associated with CRP and IL-6. The inverse association of omega-3 fatty acids with CRP and IL-6 was not modified by demographics, body mass index, smoking, LDL-C, or statin use [47]. In addition, within the scope of cardiovascular diseases, a recent report by Chang et al. [49] confirmed that a higher omega-6/omega-3 fatty acid ratio is significantly associated with shorter leukocyte telomere lengths and an increased CAD risk among a Singaporean Chinese population. These associations were mainly driven by elevated plasma total omega-3 PUFAs, especially EPA and DHA. Interestingly, they also showed that the effect of the interaction between rs529143 (an intergenic single nucleotide polymorphism) and plasma total omega-3 PUFAs and DHA on leukocyte telomere length was statistically significant beyond the genome-wide threshold [49].

Currently, the use of leukocyte telomere length as a biological marker of the aging process does not raise any doubts and is used in many studies on dietary habits [30], chronic kidney disease [54], and diabetes mellitus [99]. Omega-3 PUFAs can reduce inflammation and decrease oxidative stress [100,101] and can thereby buffer telomeres from their damaging effects. It is also possible that blood levels of PUFAs, as well as a lower omega-6/omega-3 PUFA ratio, may not only be an antioxidant body indicator but also a possible indicator of proinflammatory cytokine production. In the first reports, PUFAs, and especially total omega-3 fatty acids, were independently associated with lower levels of proinflammatory markers, such as IL-6, IL-1ra, TNF, and CRP, and with higher levels of anti-inflammatory markers, such as soluble IL-6r, IL-10, and transforming growth factor beta (TGFβ), independent of confounders [102]. Later, it was clearly shown that plasma omega-3 fatty acids were inversely associated with CRP, IL-6, and TNFα, and that plasma omega-6 fatty acids were inversely associated with CRP, IL-6, and fibrinogen. Monounsaturated fatty acids were inversely associated with CRP and IL-6. Interestingly, the omega-6/omega-3 ratios exhibited the strongest positive correlations with all the markers studied [103].

In line with this early evidence, it is interesting to analyze a later study by Kiecolt-Glaser et al. [52]. The double-blind, four-month trial included 106 healthy, sedentary, overweight, middle-aged, and older adults who received 2.5 g/day omega-3 PUFAs, l.25 g/day omega-3 PUFAs, or placebo capsules. Although the group differences with respect to telomerase and telomere length were not significant, changes in the omega-6/omega-3 PUFA plasma ratios helped clarify the intervention’s impact, namely, the telomere length increased with decreasing omega-6/omega-3 PUFA ratios. The data suggest that lower omega-6/omega-3 PUFA ratios can impact cell aging. It should also be stressed that supplementation significantly lowered oxidative stress, as measured by F2-isoprostanes. The estimated geometric mean log-F2-isoprostane values were 15% lower in the two supplemented groups compared to the placebo. Moreover, in their previous four-month randomized, controlled trial, serum IL-6 decreased by 10% and 12% in the low- (1.25 g/day) and high- (2.5 g/day) dose omega-3 PUFA groups, respectively, compared to a 36% increase in the placebo group. Similarly, the low- and high-dose omega-3 PUFA groups showed modest changes (0.2% and −2.3%, respectively) in serum TNFα, in contrast to the 12% increase in the control group [104]. These two consecutive publications by the research group from the Ohio State University College of Medicine are some of the first studies pointing to the role of omega-3 fatty acids in modulating telomere aging through oxidative and inflammatory stress.

In the context of the above results, the research involving 2284 participants of the Nurses’ Health Study should be mentioned. In the study, the authors noted that the intake of PUFAs was negatively associated with the leukocyte telomere length [48], although these results have been disputed by others [105], pointing out that the Cassidy et al. did not distinguish between omega-6 and omega-3 PUFAs in the interpretation of their data. However, considering that shorter telomeres may represent a potential marker of the cumulative burden of oxidative stress and inflammation, the authors found that dietary fiber intake is positively associated with leukocyte telomere length in the populational study. In particular, the consumption of cereal and whole-grain fiber suggests that a diet rich in plant products may benefit telomere length through anti-inflammatory and antioxidant mechanisms. Interestingly, an analysis of other factors showed a relationship between an increased consumption of the antioxidant vitamin E and leukocyte telomere length, although it was not statistically significant. No significant associations were found between vitamin D consumption, fruit and vegetable consumption, smoking, physical activity, or postmenopausal hormone use [48].

Several studies have suggested that leukocyte telomere length is a dynamic factor modifiable by lifestyle practices [30]. Sun et al. [106] reported that a healthy lifestyle is associated with a longer leukocyte telomere length in US women and that smoking, low levels of physical activity, unhealthy dietary patterns, and alcohol consumption may be associated with the shortening of the telomeres. It also seems that among the above-mentioned risk factors, dietary patterns and nutritional content—including saturated fatty acids—are the most important [51,107,108,109]. Several investigations have attempted to find the impact of nutrients and bioactive components on telomere length [110]. Some studies indicated a relationship between consumed foods, such as fruit or vegetables [109,111] and dietary fiber [48], and telomere length. In this context, another study in a group of 287 participants (55% males; 6–18 years) showed that the consumption of legumes and the intake of PUFAs were associated with leukocyte telomere length, while a higher level of cereal consumption was associated with shorter telomeres. Moreover, in the same study, a lower level of white bread consumption in the diet of Spanish children and adolescents was associated with longer telomeres [30], which may indicate a negative effect of the glycemic index on leukocyte telomere length. Observational and interventional studies have suggested that an intake of high glycemic-load foods contributes to increases in oxidative stress indicators [112]. Interestingly, in the study by García-Calzón et al., the dietary total antioxidant capacity (TAC) value was calculated by adding the TAC values from the ferric-reducing antioxidant power assay of each type of food, as previously reported [113], and was expressed as TAC in mmol/100 g food. The authors concluded that longer telomeres were associated with a higher dietary TAC [30]. Determining an appropriate dietary TAC may to some extent explain the associative problems of certain dietary components with leukocyte telomere length, while dietary patterns understood as a specific eating style, e.g., the Mediterranean diet (MD) as a whole, have been shown to be positively correlated with telomere length. Many epidemiological studies have reported the beneficial effects of the MD, which is based on its main essential ingredients: (1) long-chain omaga-3 fatty acids from fresh fish, canola oil, and soybean oil, as well as from the consumption of the succulent purslane, almonds, and walnuts, and (2) polyphenols, including flavonoids from grains, vegetables, fruits, extra-virgin olive oil, and from beverages, such as red wine, tea, chocolate, and coffee [114]. In fact, the MD, widely considered a model of healthy eating, demonstrated its protective role in leukocyte telomere length in a study of 4676 healthy US women under the Nurses’ Health Study program [115] and in a study of 217 elderly Caucasian subjects living in Campania (Southern Italy) [116]. Interestingly, the work of the Italian study showed that the eating habits closest to the traditional MD (the group with the highest MD adherence) were statistically significantly associated with reduced levels of CRP, IL-6, and TNFα in plasma in comparison to the group with the lowest MD adherence. Another epidemiological study involved three groups of participants with different diets: (1) the MD supplemented with olive oil (n = 211), (2) the MD supplemented with mixed nuts (n = 170), and (3) a low-fat diet as a control group (n = 140). The two MD groups (groups 1 and 2) showed improvement in their obesity rates and an increase in telomere lengths observed after 5 years of intervention [30].

At this point, it should be noted that the oxidative stress occurring in cells is characterized by the excessive production of ROS with a concomitant reduction of antioxidant defense mechanisms. Quantifying oxidative damage products in biological systems is important to understand the role of free radicals in telomere maintenance. Lipids, which undergo peroxidation, are major targets of free radical attack; therefore, some of the chemically and metabolically stable oxidation products are useful in vivo biomarkers of lipid peroxidation. F2-isoprostanes are a series of chemically stable compounds generated from the peroxidation of unsaturated fatty acids in membrane phospholipids independently of the cyclooxygenase enzyme. The quantitation of F2-isoprostanes is presently thought to provide a reliable and useful assessment of in vivo lipid peroxidation and oxidative stress [117]. Therefore, the elevated levels of F2-isoprostanes’ markers may indicate the susceptibility of telomeres to excessive attrition due to oxidative stress. Some of the first studies using F2-isoprostane measurements as an indicator of oxidative stress in the context of omega-3 fatty acid administration were those by Mori et al. [101,118], which showed that providing omega-3 fatty acid-containing fish oil and EPA or DHA as a dietary supplement to Type 2 diabetic patients suppresses oxidative stress [93,110]. In two subsequent 6-week, placebo-controlled intervention studies, the authors determined whether EPA or DHA supplementation affected plasma F2-isoprostane in overweight dyslipidemic men (study A) and treated hypersensitive Type 2 diabetic patients (study B); the patients were randomized to receive 4 g daily of EPA, DHA, or olive oil as a control. The post-intervention plasma F2-isoprostane levels were significantly reduced by EPA (24% in study A and 19% in study B) and by DHA (14% in study A and 23% in study B) relative to the control group [119].

All of these groundbreaking studies aroused interest in the topic during the following years, with a particular emphasis on the perinatal period. In a randomized, controlled study, 98 Australian atopic pregnant women were given a fish oil supplement (4 g/day) or a placebo (olive oil) until delivery. The authors reported that the leukocyte telomere lengths of the circulating cells of their offspring were not significantly different between the intervention groups, either at birth or at the age of 12 years [120]. Importantly, the supplementation significantly reduced the level of F2-isoprostanes in the umbilical cord plasma of newborns, but these differences were no longer visible at the age of 12. In line with these data was the previous report by Barden et al. [121], in which 83 pregnant atopic women were randomized to receive 4 g daily of either fish oil or olive oil capsules in a controlled trial from 20 weeks of gestation until delivery. A maternal fish oil supplementation lowered cord blood plasma and urinary F2-isoprostanes in neonates at a high risk of atopy [121]. However, a subsequent study suggested that the effect of omega-3 fatty acids on telomere length may be due, in part, to a reduction in oxidative stress. The effect of omega-3 fatty acids on telomere length was studied in a double-blind, placebo-controlled trial investigating CKD. Eighty-five CKD patients were randomized into two groups, given omega-3 fatty acids (4 g) or a control (4 g olive oil) daily for 8 weeks. The omega-3 fatty acid supplementation in patients with CKD was associated with an increase in the neutrophil telomere length, which was corrected for the neutrophil count. The post-intervention telomere length was negatively associated with plasma F2-isoprostanes [54].

In an interesting study, Yeates and colleagues investigated whether telomere length at 5 years of age is related to a prenatal exposure to fish-derived compounds that are either health-promoting (omega-3 fatty acids) or harmful (methylmercury), which was determined with respect to the Seychelles, a country where the level of fat consumption from fish is typically high [56]. The authors assessed both the omega-3 fatty acid status and the telomere length in blood samples collected from mothers (at 28 weeks of gestation and at parturition) and from infants, while methylmercury exposure was measured in both maternal and infant hair samples. No significant associations were found between telomere length and blood omega-3 fatty acid status or methylmercury in hair [56]. On the other hand, in the same study, the authors observed that a higher family socioeconomic stress score, measured at 9 months of age, was associated with a longer telomere length in the infants at birth. In contrast, studies of the animals in an induced GDM rat model, given either omega-3 PUFAs (fish oil) or safflower for 11 months, showed that omega-3 PUFAs decreased oxidative stress and inflammation in the liver. Moreover, omega-3 PUFAs reduced the long-term risk of diabetes in the offspring of GDM rats by delaying the shortening of hepatic telomeres and modulating liver metabolism [61].

Data on the relationship between telomere length and the use of aspirin, an anti-inflammatory agent, and an antioxidant, are inconsistent and depend on the type of cell studied [122]. The effects of aspirin alone, EPA + DHA, and the combined effects of aspirin and EPA + DHA treatment on telomerase activity have been explored in a limited number of 30 adults with diabetes mellitus [99]. All the participants ingested non-enteric 81 mg aspirin and a dose of 4000 mg of over-the-counter fish oil with mean concentrations of DHA and EPA in the study capsules of 406 ± 42 mg/mL and 330 ± 46 mg/mL, respectively. Bearing in mind that type 2 diabetes is associated with aging and the shortening of telomere length, it has been shown that EPA and DHA ingestion alone increased telomerase activity, and that a decrease occurred with the addition of aspirin consumption. These results suggest that aspirin has an adverse effect on aging in diabetics who have a relatively high intake of EPA and DHA [99].

A very important aspect that often escapes researchers’ attention is the fact that the essential unsaturated fatty acids, LA and ALA, are desaturated and elongated into their long-chain metabolites—arachidonic acid (AA), EPA, and DHA—by D6 and D5 desaturases, respectively, which in turn are the precursors of the eicosanoid lipid mediators, namely, prostaglandins (PGs) and leukotrienes (LTs), lipoxins (LXs), epoxyprostanoids, resolvins, protectins, maresins, and isoprostanes. Before a reaction occurs, PUFAs are usually released from membrane phospholipids mainly by phospholipase A2 (PLA2) action. The release of non-esterified PUFAs from membrane lipids can be enhanced by specific physiological stimuli (e.g., adrenaline, angiotensin II, and certain antibody–antigen complexes) or non-specific pathological conditions. From AA, two series of PGs and TXs and four series of LTs are formed, while three series of PGs and TXs and five series of LTs are synthetized from EPA. PGs, TXs, and LTs are generally proinflammatory in nature, except that the three series of PGs and TXs and the five series of LTs are less proinflammatory than those formed from AA. Lipoxins are derived from AA, resolvins from EPA and DHA (resolvins of E series from EPA and resolvins of D series from DHA), and protectins from DHA. DHA also forms a precursor to maresins. LXs, resolvins, protectins, and maresins are anti-inflammatory compounds that antagonize the proinflammatory actions of PGs, TXs, and LTs [123]. Thus, there is a balance maintained between the pro- and anti-inflammatory compounds formed from AA, EPA, and DHA under physiological conditions. Given this delicate balance between anti- and pro-inflammatory lipid mediators, it is important to identify and characterize the mediators derived from omega-3 fatty acids, including their synthesis pathways and their regulation. Thus, it is important to appreciate the complexity of the lipid mediator system in physiology and pathology, as it highlights the potential effects of diet and nutrition on human health. It is also possible that an adequate supply of fatty acids in the diet that changes the omega-6/omega-3 fatty acid ratio in lipid membranes may be an important element in maintaining the pro and anti-inflammatory balance in the cell [124].

### Direct Effect of Omega-3 Fatty Acids on Telomere Length

In addition to the reports indicating the anti-inflammatory and antioxidant effect of omega-3 fatty acids in the context of telomere protection, there are studies that show a diversified effect of omega-3 fatty acids on telomerase, a ribonucleoprotein that adds the tandem arrays of TTAGGG repeats to telomere ends. In humans, the enzyme is composed of hTR (human telomerase RNA component), TP1 (telomerase-associated protein 1), and hTERT (human telomerase reverse transcriptase), the last of which plays a key role in telomerase activation. Telomerase was thought to be expressed only in germ cells, stem cells, and cancer cells [125]. Indeed, most human somatic cells do not actively express this enzyme. This holds true with the exception of peripheral blood mononuclear cells, which can upregulate telomerase activity when activated [126,127]. Eitsuka et al. [128] found that fatty acids (C18–C22) directly inhibit telomerase activity. The IC50, the concentration at which 50% of telomerase activity is inhibited, indicated that the inhibitory potency of fatty acids increases with the number of double bonds [129]. Accordingly, PUFAs, such as EPA and DHA, can strongly prevent telomerase’s enzymatic activity. A Lineweaver–Burk plot revealed that EPA is a competitive inhibitor relative to the telomerase substrate primer, implying that fatty acids may interact with the primer-binding site of telomerase. Moreover, they demonstrated that physiological concentrations of EPA and DHA (≤50 μM) downregulate hTERT and c-Myc mRNA via PKC inhibition, thereby repressing telomerase activity. These results indicate that fatty acids, especially EPA and DHA, not only inhibit the enzymatic activity of telomerase directly but also downregulate telomerase at the transcriptional level [128]. Unexpectedly, others have also reported that both PUFAs and fish oil effectively inactivated testicular telomerase and inhibited c-Myc-mediated telomerase reverse transcriptase expression, whereas omega-3 PUFAs rather than omega-6 PUFAs protected the liver and testes against telomere shortening within the ranges of 13–25% and 25–27%, respectively [59]. This finding is probably associated with the “double-edged sword” property of telomerase. Telomerase is canonically responsible for telomere length maintenance, though its activation may favor tumorigenesis. Nevertheless, both omega-3 and omega-6 PUFAs significantly reduced telomerase activity independent of telomere length. Numerous scientific papers have indicated the possibility of modulating TERT expression through epigenetic regulation. In cancers, DNA hypermethylation with the TERT promoter is a prevalent telomerase-activating mechanism that can act independently of or in conjunction with promoter mutations [130]. Liu et al. [50] found that erythrocyte DHA content was decreased in a cohort of 46 obese vs. 46 lean preschool children, and it had a positive association with leukocyte telomere length. Unfortunately, no association between erythrocyte DHA and DNA methylation of the TERT promoter was shown, although DHA functions to regulate DNA and histone methylation by affecting methyl group metabolism [50].

At this point, it is also worth mentioning an interesting theory formulated by Ponnusamy et al. [131] on the regulation of telomere length by fatty acid elongases, which are enzymes that mediate the elongation of fatty acids into very-long-chain fatty acids. In the context of omega-3 fatty acids, elongases are involved in the formation of EPA and DHA from their precursor ALA. Ponnusamy et al. [131] determined that the deletion of fatty acid elongase 3 not only leads to a decreased level of very-long-chain fatty acids in yeast cells but also reduces telomere length. The authors postulated that this phenomenon is mediated by telomere binding and the protective function of the YKu 70/80 protein, as an immunoprecipitation assay showed that its function was reduced in elo3Δ cells, whereas its nonhomologous end-joining function was not altered. The Ku70/80 heterodimer is one of the most abundant nuclear factors that inter alia protects telomere ends from degradation and regulates telomere length, possibly through a functional interaction with telomerase [132]. Similar results were obtained by Askree et al. [133], where the deletion of the gene encoding elongase 3 in yeast led to shorter telomeres compared to wild-type cells [133]. The presented results in yeast are supported by the results obtained several years later in rats [60]. Rats fed fish oil rich in omega-3 fatty acids, leading to abundant tissue levels of omega-3 fatty acids, expressed higher levels of Ku70 transcripts in the liver. Thorough research is needed to evaluate the interactions between omega-3 fatty acids, elongases, and Ku 70/80 in terms of telomere protection.

Lastly, what if omega-3 fatty acids affect telomere length in the most obvious way, namely, by slowing down the rate of cell proliferation? Bartram et al. [134,135] observed that daily supplementation with omega-3 fatty acids decreased the proliferation of rectal cells biopsied from healthy volunteers [134,135]. Interestingly, PG E2 release from rectal biopsy specimens (435.5 vs. 671.5 pg/mg wet tissue; *p* < 0.05) was significantly lower in the group given fish oil vs. that given corn oil [135]. A similar effect was observed in rats, where the addition of omega-3 fatty acids to fodder resulted in a low proliferation rate of intestinal mucosal cells [136]. Such a theory, however, needs further examination, but the results support the hypothesis that dietary fish oil may protect against colon cancer.

## 5. Limitations

It should be remembered that the substantial evidence presented thus far on the effects of omega-3 fatty acids on leukocyte telomere length has several potential limitations, including small sample sizes that reduce the statistical power to find differences, and long blood storage times resulting in delayed tissue processing and possible oxidation. Furthermore, the measurements of telomere length in leukocytes may not reflect the telomere length dynamics in other tissues, and telomere length dynamics may differ among other ethnicities; hence, the results may not be generalizable. From a methodological point of view, some scientists postulate that a quantitative FISH protocol using a specific fluorescent probe hybridized to telomeric repeats would be more accurate than the qPCR approach that is commonly used. In addition, many studies rely on a single measurement of telomere length and therefore cannot investigate inter-individual variation in telomere length over extended periods of time; the absence of serial measurements may result in a limited ability to detect associations. Finally, the cross-sectional study design limits the potential to discern causative relationships. There is also the possibility of a measurement error as the participants and/or their parents self-reported their habits in the food frequency questionnaires. As in any observational study, measurement bias in self-reported variables is inevitable.

## 6. Conclusions

**Figure 1:**
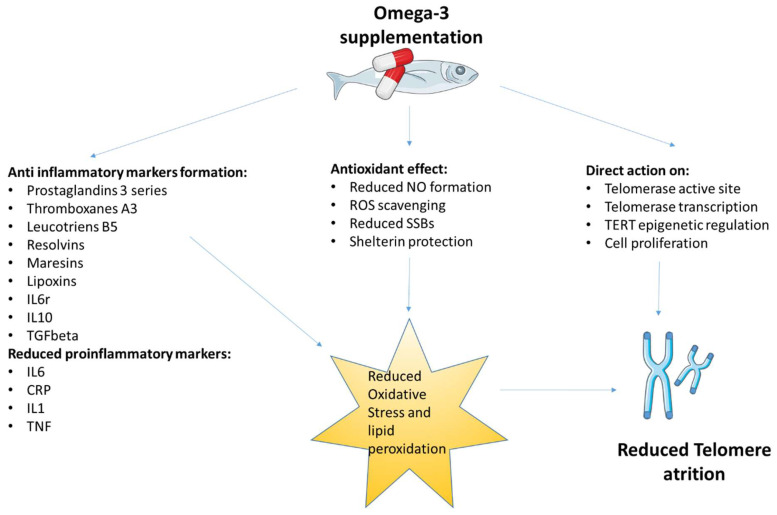
Indirect and direct effects of omega-3 fatty acid supplementation on telomere attrition.

While the results of the presented cross-sectional and randomized human and rodent studies are not entirely consistent, the overwhelming number of them have demonstrated the beneficial effects of omega-3 fatty acids on telomere length (Figure 1). The factors that are strongly associated with accelerated telomere shortening and dysfunction are oxidative stress and inflammation. The ability of omega-3 fatty acids to reduce these negative effects is related not only to their well-documented beneficial effect on a number of ‘lifestyle’ diseases but also to their beneficial effects on telomere biology, which have both been raised in this review. The use of omega-3 fatty acids to reduce accelerated telomere attrition and, consequently, counteract premature aging and reduce the risk of age-related diseases raises high hopes. However, discrepancies in the presented results still indicate the need for a careful evaluation of the type of omega-3 fatty acids, their origin, dose and the timing of administration, as well as age, gender, regional and ethnic diversity, and health status.

## Figures and Tables

**Table 1:** Studies documenting a link between omega-3 fatty acid intake and telomere length in humans.

Author, Year [Ref]	Study Design	Examined Group	Omega-3 Fatty Acids Adjustment	Reported Effect on Telomere Length *
Farzaneh-Far et al. [47]	Prospective cohort study	608 U.S. men and women with coronary artery disease, follow-up of 5 years	Quartiles of baseline sum of omega-3 fatty acids (EPA and DHA) in whole blood estimated by gas chromatography (GC)	Inverse associations between levels of omega-3 fatty acids and leukocyte telomere length
Cassidy et al. [48]	Cross-sectional study	2284 U.S. women, 43–69 years old	Marine omega-3 fatty acids and ALA intake per day, estimated based on questionnaire	No association between marine omega-3 fatty acids, ALA, and leukocyte telomere length
Chang et al. [49]	Prospective cohort study	711 Chinese patients with nested coronary artery disease and 638 healthy controls	Plasma levels of ALA, EPA, and DHA quantified using mass spectrometry	Positive correlation between EPA and DHA levels and leukocyte telomere length
Liu et al. [50]	Cross-sectional study	64 children with obesity and equal numbers of normal weight children, 3–4 years old	Erythrocyte levels of ALA, EPA, eicosatetraenoic acid, and DHA measured with GC	Significant association between DHA levels and leukocyte telomere length
Freitas-Simoes et al. [51]	Cross-sectional study	344 Mediterranean people (63–79 years old, 68.6% women)	Erythrocyte levels of ALA, EPA, and DHA measured with GC	No association between level of ALA, sum of EPA and DHA, and leukocyte telomere length, measured by quantitative fluorescence in situ hybridization (FISH)
Kiecolt-Glaser et al. [52]	Randomized controlled trial	106 U.S. men and women, 40–85 years old, receiving 2.5 g omega-3 PUFAs, 1.25 g omega-3 PUFAs, or placebo capsules mirroring the proportions of fatty acids in an average American diet per day for 4 months	Change in the level of omega-6 and omega-3 fatty acids in blood plasma	No significant changes in telomere length between groups. Telomere length increased with decreasing omega-6:omega-3 fatty acid ratio
O’Callaghan et al. [53]	Randomized parallel-group pilot trial	33 Australian men and women, >65 years old, suffering from mild cognitive impairment, receiving a supplement rich in EPA (1.67 g + 0.16 g of DHA; n = 12), a supplement rich in DHA (1.55 g + 0.40 g of EPA; n = 12), or LA (2.2 g; n = 9) per day for 6 months	Erythrocyte DHA level	Telomere shortening was greatest in the LA group (d = 0.21) compared to the DHA (d = 0.12) and EPA (d = 0.06) groups, but results were statistically underpowered. Increased erythrocyte DHA levels were associated with reduced telomere shortening (r = −0.67; *p* = 0.02) in the DHA group.
Barden et al. [54]	Randomized parallel-group pilot trial Double-blind placebo-controlled trial	85 men and women, 25–75 years old, suffering from chronic renal impairment, receiving omega-3 fatty acids (4 g), or CoQ (200 mg), or both supplements, or control (4 g of olive oil) per day for 8 weeks	None	Telomere length corrected for neutrophil count was increased after omega-3 fatty acids
See et al. [55]	Randomized parallel-group study	98 pregnant atopic women receiving 4 g of omega-3 PUFAs or an equal amount of olive oil from 20 weeks’ gestation until delivery; offspring of examined women	Erythrocyte omega-3 fatty acids level measured with GC	Maternal supplementation with omega-3 fatty acids did not affect offspring’s telomere length at birth or at 12 years, with no changes over time
Yeates et al. [56]	Cross-sectional study	229 mothers (mean age: 27.2 years, range: 15–42 years) and their children	Level of omega-3 fatty acids in blood of mothers (at 28 weeks of gestation and at delivery) and children (at 5 years of age) determined using Gas chromatography/mass spectrometry (GC/MS)	Omega-3 fatty acid level was not associated with telomere length of the mother or child or with telomere length attrition rate
Liu et al. [57]	Cross-sectional study	274 mothers (mean age: 31.96 ± 3.70) and their children	Level of omega-3 fatty acids in maternal erythrocytes and cord blood, determined using GC	Low concentrations of DPA and total omega-3 fatty acids in maternal erythrocytes and low concentrations of cord blood DHA were associated with shortened telomere length in cord blood cells. High concentrations of ALA, eicosatrienoic acid (EA), and DHA in maternal erythrocytes were associated with shortened telomere length in the placenta.

* For most studies, telomere length was measured using real-time PCR based on the method designed by Cawthon [58]; if not, information about the technique used is given in the table.

**Table 2:** Studies documenting a link between omega-3 fatty acid intake and telomere length in rodents.

Author, Year [Ref]	Study Design	Examined Group	Omega-3 Fatty Acids Adjustment	Reported Effect on Telomere Length *
Chen et al. [59]	Animal study	8-week-old male mice (n = 5) were assigned to groups receiving D-galactose to induce aging; this included a positive D-galactose group and groups receiving 400, 200, and 100 mg of fish oil per kg of body weight per day or 120, 60, and 30 mg of DHA per kg of body weight per day for 2 months.	None	Omega-3 fatty acids protected the liver and testes against telomere shortening within the range of 13–25% and 25–27%, respectively.
Varela-Lopez et al. [60]	Animal study	72 male rats were assigned to three groups and fed—from weaning until 24 months of life—fodder differing in fat source (virgin olive oil, sunflower oil, or fish oil rich in DHA and EPA); the amount of fat fulfilled the standard requirement of a rat’s diet.	Liver fatty acid profile determined with GC	24-month-old rats receiving a diet rich in fish-based omega-3 fatty acids exhibited the longest liver telomeres compared to 6-month-old animals receiving the same diet and to animals fed virgin olive oil and sunflower oil
Gao et al. [61]	Animal study	10 healthy rats and 34 suffering from gestational diabetes mellitus (GDM) were divided into three groups: offspring fed soybean oil, adequate offspring fed fish oil (rich in omega-3 fatty acids), and omega-3 PUFA-deficient offspring fed safflower oil until 11 months old. Rats belonged to both sexes.	The level of ALA in liver was determined with high-performance liquid chromatography–quadrupole-time of flight mass spectrometry (HPLC-QTOF-MS)	The liver telomere length of rats suffering from GDM was nearly improved (with a nonsignificant tendency) by supplementation with omega-3 fatty acids, compared with non-supplemented GDM rats.

* For all studies, telomere length was measured using real-time PCR based on the method designed by Cawthon [58].
